# Case of Pulmonary Apoplexy

**Published:** 1858-06

**Authors:** O. W. Newell

**Affiliations:** Plano, Kendall Co., Ill.


### CASE OF PULMONARY APOPLEXY.

BY O. W. NEWELL, PLANO, KENDALL CO., ILL.

Messrs. Editors: The following case is one of deep interest to me, and over which may still hang a doubt. I send it to the *Journal* for publication and remarks:

On the morning of Nov. 18th, '57, I was called to the residence of Mr. B., whose wife had died very singularly the night previous, as was supposed of Cynanche Trachalis. The deceased was a lady of thirty, of full plethoric habit, was enciente of about six months. The night previous to her demise she ate a hearty supper, feeling as well as usual excepting a slight cold, went to bed at nine o'clock. About fifteen minutes before 11, she awoke and requested her husband to get up as she felt very strange, to use her own words, "I feel as though I was scared." She requested him to go for a physician. A Madam Lobelia, who lived by was called in, whose mature judgment decided croup, and down went a teaspoonful tinc. lobe. in a table spoonful Olive oil. As vomiting commenced, she sank back into the arms of her husband and died without a struggle living about fifty minutes after she awoke. I saw her about ten hours after she died; there was no distortion of the features, pupils appeared natural, her face, neck, breast and upper extremities, were of a dark, purple color, body warm, and a white froth issuing from the mouth and nose. Not feeling satisfied that she died from croup, I remarked to my student, Mr. Dowd, that all did not appear right, as she bore the appearance of vegetable poisoning, and I was resolved on an examination. I then learned her history from her husband and others; found she was enciente with her first child, that she and her husband were well pleased, considering it a triumph, as they had been married a number of years. I then inspected all medicines and other things about the house and carried a number of articles home to my office for examination. I then insisted on a post mortem of the bronchial tubes which was granted. I found them in a perfectly healthy state not even inflamed and perfectly free from foreign substance, phlegm or mucus. Of course, then, all was dark. I could then account for the death under the circumstances only by apoplexy of the lungs or by poison. I insisted on a further examination but was not permitted to do so. On the morning of the 19th, Mr. B. came in saying, that as her friends and others did not feel satisfied as to the cause of her death, I was permitted to make further examination. This morning, the dark, purple appearance had left the face, there was no more frothing from the mouth and nose. I then opened to the lungs, external viscera healthy; on removing the sternum the lungs were found full and engorged with dark blood on the outside, and a bright scarlet in the inner cells. There was some cracking sensation on pressure. I could only inflate the upper portion. On laying them open, in some parts minute particles of coagulated blood could be scraped out with the scalpel, leaving the cell empty; in other parts of a bright scarlet color, and on being cut bled freely. Every portion of the lungs was completely engorged. Otherwise all bore a healthy appearance; no tubercles, no solidified portion, no inflammation of the smaller pipes of the bronchial tubes, but a general effusion of blood in the pulmonary parenchyma. The heart and arteries were all sound and healthy. There was no blood in the arteries, a very little in the heart and in the pulmonary artery.

The liver, spleen, kidneys, abdominal viscera, uterus, and foetus were all evidently in a healthy condition. If there was anything about any organ that bore any pathological condition aside from the lungs, it escaped my notice. There was no corrosion about the mouth or æsophagus; the stomach contained a small portion of partly digested food and some fluid—about eight ounces in all—on the top of which floated the oil previously taken, nothing about it bearing marks of disease. Taking all things as I found them, I can not account for death from any other source than apoplexy of the lungs. Death by opium will cause the same ecchymosed condition of the skin, and in "Taylor on Poisons," (page 740), a case is reported of a similar condition of the lungs, and also, (on page 523) by prussic acid.

Gentlemen, I diagnosed the case as one of pure apoplexy of the lungs. Am I right in my diagnosis?

